# A Pilot Trial Assessing Urinary Gene Expression Profiling with an mRNA Array for Diabetic Nephropathy

**DOI:** 10.1371/journal.pone.0034824

**Published:** 2012-05-18

**Authors:** Min Zheng, Lin-Li Lv, Yu-Han Cao, Hong Liu, Jie Ni, Hou-Yong Dai, Dan Liu, Xiang-Dong Lei, Bi-Cheng Liu

**Affiliations:** 1 Institute of Nephrology, Zhong Da Hospital, Southeast University School of Medicine, Nanjing, China; 2 CT Bioscience, Chang Zhou, China; California Pacific Medicial Center Research Institute, United States of America

## Abstract

**Background:**

The initiation and progression of diabetic nephropathy (DN) is complex. Quantification of mRNA expression in urinary sediment has emerged as a novel strategy for studying renal diseases. Considering the numerous molecules involved in DN development, a high-throughput platform with parallel detection of multiple mRNAs is needed. In this study, we constructed a self-assembling mRNA array to analyze urinary mRNAs in DN patients with aims to reveal its potential in searching novel biomarkers.

**Methods:**

mRNA array containing 88 genes were fabricated and its performance was evaluated. A pilot study with 9 subjects including 6 DN patients and 3 normal controls were studied with the array. DN patients were assigned into two groups according to their estimate glomerular rate (eGFR): DNI group (eGFR>60 ml/min/1.73 m^2^, n = 3) and DNII group (eGFR<60 ml/min/1.73 m^2^, n = 3). Urinary cell pellet was collected from each study participant. Relative abundance of these target mRNAs from urinary pellet was quantified with the array.

**Results:**

The array we fabricated displayed high sensitivity and specificity. Moreover, the Cts of Positive PCR Controls in our experiments were 24±0.5 which indicated high repeatability of the array. A total of 29 mRNAs were significantly increased in DN patients compared with controls (p<0.05). Among these genes, α-actinin4, CDH2, ACE, FAT1, synaptopodin, COL4α, twist, NOTCH3 mRNA expression were 15-fold higher than those in normal controls. In contrast, urinary TIMP-1 mRNA was significantly decreased in DN patients (p<0.05). It was shown that CTGF, MCP-1, PAI-1, ACE, CDH1, CDH2 mRNA varied significantly among the 3 study groups, and their mRNA levels increased with DN progression (p<0.05).

**Conclusion:**

Our pilot study demonstrated that mRNA array might serve as a high-throughput and sensitive tool for detecting mRNA expression in urinary sediment. Thus, this primary study indicated that mRNA array probably could be a useful tool for searching new biomarkers for DN.

## Introduction

Diabetic nephropathy (DN) is a serious complication of diabetes with high morbidity and mortality. It has become the most common cause of end-stage kidney disease in the world [1]. Therefore, the early identification of people at great risk of DN is of the utmost importance.

Initial increase in albumin excretion rate (AER) has been traditionally linked to a subsequent decline in glomerular filtration rate (GFR) and microalbuminuria (MA) has been established as an early biomarker of DN in clinic [2]. However, approximately 20% of people with type 2 diabetes develop at least stage 3 CKD, defined as an estimated GFR (eGFR) less than 60 ml/min/1.73 m^2^, while remaining normoalbuminuric. This discordance between changes in GFR and AER has resulted in a search for new markers that identify people with diabetes who are at risk of declining GFR independent of progressive increases in AER [3].

In the past decade, evidence from in vitro experiments and pathological examinations to epidemiological studies, has shown that pathogenetic mechanism of DN involved complex molecular pathways. The key molecules and pathways included inflammation [4], epithelial-to-mesenchymal transition [5], extra-cellular matrix deposition, tubular cell and podocyte injury [6], [7]. Target screening of those related molecules might provide us an efficient strategy for novel biomarker discovery.

Recently, RNA extraction from urinary sediment combined with real-time quantitative PCR has emerged as a novel strategy for identifying biomarkers with kidney disease. Preliminary studies have suggested that the determination of urinary mRNA levels might be valuable in monitoring the progression of renal disease [8], [9]. Our previous studies have also indicated the potential application of urinary mRNA detection in searching novel biomarkers of kidney disease [10], [11]. However, considering the numerous molecules involved in DN development, a high-throughput platform with efficient detection capacity is needed. Gene expression array has emerged as a promising strategy for analyzing multiple gene expression in parallel [12]. Among the various array formats, PCR array is the most reliable tools for analyzing the expression of a focused panel of genes [13]. The arrays can be run efficiently in parallel, enabling studies on the large populations of molecules involved in disease development that are necessary for marker discovery and validation.

In this study, a target PCR array was constructed with the aim to develop a simple, easy-to-use tool for differential mRNA screening with urinary sediment. And the PCR array was applied to profile the expression of a panel of mRNAs relevant to pathways involved in DN pathophysiology. The screening with such platform might provide important clues as for candidate gene markers for further validation. To our best knowledge, this is the first study to apply PCR arrays to analyze urinary mRNA expression with aims to screening promising genes for DN diagnostics.

## Results

### 1 Characterization of Real Time PCR Array

#### 1.1 Sensitivty

We assessed sensitivity of PCR array in terms of positive call rate, which means the percentage of genes detectable in an array. Figure S1 shown the percentage of positive calls (the percentage of genes with Ct<38) against different samples. The results indicate that the PCR array system achieved greater than 80 percent positive calls with all samples, and 5 out of 9 over 90% positive calls, 8 out of 9 over 85%.

#### 1.2 Specificity

Figure S2 shown the melting curve analysis which was used to assess the specificity of the array. As displayed in the figure, a single product of the predicted size from each reaction without secondary products such as primer dimers indicated the high specificity of PCR array.

#### 1.3 Reproducibility

The PPC contains artificial DNA template which has no homology to the DNA of the samples and a validated primer pair which can be used to check plate-to-plate and run-to-run reproducibility of the array. Both plate-to-plate or run-to-run variation will result in PPC Ct variation. As shown in Figure S3, the Cts of PPC in our experiments were 24±0.5. The results demonstrated that high degree of plate-to-plate and run-to-run reproducibility could be obtained.

### 2 Clinical Characteristics of Enrolled Subjects

The primary clinical and laboratory characteristics of the study subjects are summarized in Table 1. The age of participants in the control group was lower than DN patients. DNII group had a significant increase in urinary albumin excretion, serum creatinine, BUN and a decrease in eGFR compared with DNI group and normal controls (p<0.05).

**Table 1:** Primary clinical and laboratory characteristics of the study subjects.

	Controls (N = 3)	DNI (N = 3)	DNII (N = 3)
Age (years)	52.00±13.11	75.0±8.89#	66.38±8.08#
Sex (male/female)	2/1	3/0	3/0
Diabetes duration(years)	−	7.33±1.53	9.97±3.06
MBP (mmHg)	−	109±4.6	112±6.2
HbA1c (%)	−	7.16±0.69	7.18±0.57
Scr (µmol/l)	83.00±13.23	75.57±8.57	122.97±6.21*
Ualb (mg/g (Cr))	9.67±7.23	100.00±59.92	427.67±35.23*
BUN (mmol/l)	4.03±0.96	5.26±1.16	8.28±0.27*
eGFR (ml/min/1.73 m^2^)	87.84±9.03	99.22±12.72	55.16±3.60*

#DNII group and DNI group vs. controls, p<0.05.

* DNII group vs. DNI group and controls, p<0.05.

### 3 Urinary mRNA Profiles in DN Patients and Healthy Controls

Among the 88 mRNAs screened, a total of 29 mRNAs were significantly increased in DN patients compared with normal controls (p<0.05) while TIMP-1 was significantly decreased. The increased 29 mRNAs belong to different functional categories in DN development as listed in Table 2. If we divide DN group as subgroup based on the eGFR level, among those 30 mRNAs identified, 14 mRNAs (SYNPO, ACTN4, PODXL, SNAI2, UMOD, MAPK8, RBP4, MMP9, CTGF, MCP-1, PAI-1, ACE, CDH1 and CDH2) were found to show significant difference when DNI and DNII group was compared with normal controls. And other 14 (HGF, FAT1, PODXL2, MMP2, COL4A1, TWIST1, SNAI1, SMAD4, SMAD7, LAMA5, REN, NOTCH2, NOTCH3 and AGT) mRNAs show significant different level when DNII group was compared with normal controls but no difference was found when DNI group was compared with normal controls. The other 2 mRNAs (TIMP-1, cytokeratin18) show no difference when neither two subgroups were compared with normal controls.

**Table 2:** Functional categories of 29 up-regulated mRNAs in DN patients.

Categories of genes	Gene name
Tubular injury markers	RBP4, UMOD
Renin angiotensin system	ACE, REN, AGT
EMT markers	CDH1, CDH2, FAT1, LAMA5, cytokeratin18
Cytokines	CTGF, MCP-1, HGF
Podocyte markers	PODXL2, SYNPO, PODXL, ACTN4
Extra-cellular matrix related	PAI-1, MMP-2, COL4A1, MMP-9
Signal pathway related	NOTCH2, NOTCH3, TWIST1, SNAI1, SMAD4,SMAD7, SNAI2, MAPK8

Functional categories of genes up-regulated in DN patients compared with healthy controls. Among the 88 mRNAs screened, a total of 29 mRNAs were significantly increased in DN patients compared with normal controls (p<0.05).

### 4 Urinary Gene Expression between Different Stages of DN

We also evaluated the differential expression of mRNAs between different stages of DN, that is DNI and DNII group. Interestingly, 6 mRNAs shown significant difference when DNI and DNII group was compared. The mRNAs identified included CTGF (p = 0.01), MCP-1(p = 0.04), PAI-1(p = 0.04), ACE (p<0.001), CDH1 (p = 0.04) and CDH2 (p = 0.03). Moreover, those mRNAs were found to be differentially expressed when DNI and DNII group were compared with healthy controls (p<0.05).

### 5 Fold Change Expressions of mRNAs between DN Patients and Controls

**Figure 1:**
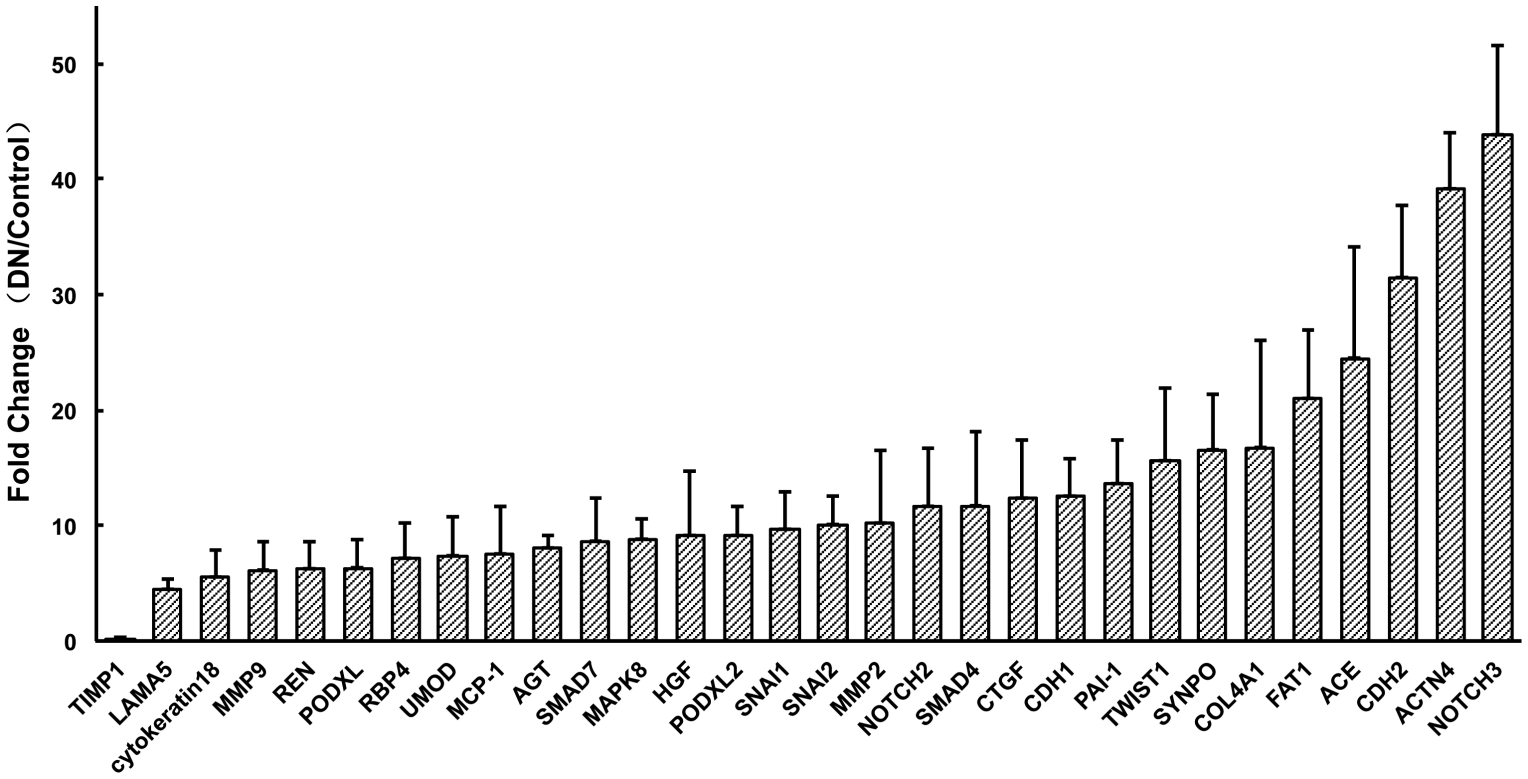
Fold change expressions of mRNAs between DN patients and controls. NOTCH3, ACTN4, CDH2, ACE, FAT1, COL4A1, SYNPO, TWIST1 were 15 fold increased and TIMP-1 was found with 15 fold decreased levels in DN group compared with normal controls.

Figure 1 shown the fold change expressions of mRNAs between DN patients and controls. In our study, a 15 fold change value was chosen as a threshold. Those mRNAs with 15 fold increased levels when DN patients were compared with normal controls included 8 genes as followings: NOTCH3, ACTN4, CDH2, ACE, FAT1, COL4A1, SYNPO, TWIST1. And TIMP-1 was found with 15 fold decreased levels in DN group compared with normal controls. No candidate was found with 15 fold change when DNI group was compared with DNII group. Fourteen mRNAs were found with 15 fold change when DNII group was compared with normal controls. Four mRNAs shown significant change in expression levels when DNI was compared with controls (NOTCH3, ACTN4, CDH2, ACE).

## Discussion

In this study, we established an mRNA profiling array specific for DN using real-time PCR technique and evaluated its performance. The array was used to screen urinary mRNA levels in DN patients. The preliminary results demonstrate its potential to define new biomarkers for kidney diseases.

Substantial effort has been spent to identify gene biomarkers that display differential expression in various disease types. However, gene expression studies in kidney disease are usually limited to the expression of a few protein-coding genes [14]. Considering the complex molecular networks involved in kidney disease, an enormous number of mRNAs may have to be examined. Array technique with high throughput is a potentially efficient approach for simultaneously analyzing large-scale gene expression profiles. Rödder et al found that a transcriptomic classifier consisting of 19 metzincins and related genes (MARGS) could discriminate biopsies from renal transplant patients with or without interstitial fibrosis/tubular atrophy by virtue of gene expression measurement [15]. In a recent study from Wu et al, cDNA microarray analysis was performed to identify gene expression changes, and highly expressed genes were evaluated as markers both in mice and human kidney samples with membranous glomerulonephropathy (MN) [16]. However, in these studies, gene arrays were applied to analyze gene expressions in renal tissue, which was an invasive assay. Urine containing cells shed from the urinary tract might provide important information about ongoing kidney damage [17], [18]. In this study, we have designed a PCR array for urinary gene expression analysis and conducted a proof-of-concept study with samples from DN patients.

The results demonstrated that the array has relatively high specificity, sensitivity and reproducibility. The high sensitivity would suggest that it could quantify mRNA expression with low amounts of extracted RNA from urinary sediment, including detection of inflammatory cytokine and receptor mRNAs that are known to be expressed at very low levels but play a critical role in DN development. The specificity and reproducibility of the system suggests the amplification of only one gene-specific product in each reaction, making the technique suitable for large-scale gene expression screening. Eighty-eight mRNAs were included in the constructed array targeting key molecules thought to be involved in DN development. The upregulated mRNAs were functionally associated with key molecular events with the development of DN including those from tubular cell and podocyte injury markers, renin-angiotensin system, EMT markers, cytokines, extra-cellular matrix related markers and signal pathway related mRNAs.

Among the mRNAs we identified, six (CTGF, MCP-1, PAI-1, ACE, CDH1 and CDH2) showed significant difference when the DNI and DNII groups were compared, suggesting these mRNAs might be involved in the development of DN and could hold promise in discriminating different stages of DN. Many previous studies have suggested that profibrotic growth factors, inflammatory chemokines and renin angiotensin system (RAS) are key players in the pathogenesis of DN [19]–[21]. CDH belongs to cell-cell adhesion molecule family and has become a marker for tracking EMT in development of kidney damage [22]. Hence, our results are compatible with dysregulation of these genes and pathways in the pathophysiology of DN. However, our findings are preliminary and need to be validated in larger cohort group of samples.

In summary, we have constructed a PCR array platform with high sensitivity, specificity and reproducibility to enable urinary mRNA profiling. In this proof-of-concept study, we demonstrated that multiple mRNAs in urine sediment can be profiled in patients and healthy controls. A few promising mRNAs with differential expression in DN patients compared with healthy controls were found suggesting biomarker candidates for further study.

## Materials and Methods

### Fabrication of PCR Array

88 potential molecules involved in the pathogenesis and progression of DN were selected as the target mRNAs. GAPDH, β2-MG, β-actin, RPL27, HPRT1 and OAZ1 were used as house keeping genes for normalization. A Genomic DNA Control (GDC) primer was also set to specifically detect non-transcribed, repetitive genomic DNA. The replicate Positive PCR Controls (PPC) was included to report on the efficiency of the polymerase chain reaction itself. The corresponding gene name was listed in part of **Primer design and selection.**

### Primer Design and Selection

Except for three genes, primers were designed to cover all transcripts of each gene. Table S1 lists the refseq accession IDs each primer pair can detect. Primer design also takes the following into considerations. All primers have similar melting temperature (Tm), primers do not contain known SNPs or locate in genomic repetitive regions. Primers are experimentally selected if they meet the following criterias: a typical amplication curve, post PCR melting curve analysis shows a single peak, single band with matched sizes analysed by agarose gel electrophoresis.

### Study Populations

All studies were approved by the Ethical Committee of Southeast University. Written informed consents were obtained from all subjects to use their urine for research purpose.

Six patients with type 2 diabetic nephropathy from Zhong Da Hospital, Southeast University School of Medicine were enrolled in this study. Diabetic nephropathy was diagnosed based on KDOQI guideline [23], that is: at least 5 years from the diagnosis of type 2 diabetes, the presence of diabetic retinopathy, elevated albumin-creatinine ratio (ACR), and the absence of clinical or laboratory evidence of other kidney disease. To evaluate progression of DN, patients were divided into two groups based on the eGFR level: DNI (eGFR>60, n = 3) and DNII (eGFR<60, n = 3). eGFR was calculated by the equation proposed by Ma et al., which was considered to be more suitable for Chinese study subjects [24]. A group of healthy volunteers (n = 3) was also enrolled in the study as a negative control. Clinical data including albuminuria, blood urea nitrogen (BUN), and serum creatinine were recorded at baseline for each of the groups.

### Collection of Urine Samples and Total RNA Extraction

A whole-stream early-morning urine specimen was collected from each study participant. Shortly after collection, the urine was centrifuged at 3,000*g for 30 minutes at 4°C. The urinary supernatant was discarded, and the remaining cell pellet was resuspended in 1.5 ml DEPC-treated PBS and was then centrifuged at 13,000*g for 5 minutes at 4°C. The pellet was then resuspended in 1.0 ml RNAiso Plus (TAKARA, Dalian, China) and was stored at −80°C until use. Total RNA was extracted according to the manufacturer’s protocol (TAKARA). The RNA concentration and purity were confirmed using the relative absorbance ratio at 260/280 on a nanodrop 2000 (Thermo, Wilmington, USA).

### Reverse Transcription

For reverse transcription, 2 µg total RNA was mixed with 8 µl 5× PrimeScript™ Buffer, 2 µl PrimeScript™ RT Enzyme Mix I, 2 µl Oligo dT Primer (50 µM), 2 µl Random 6 mers (100 µM), (TAKARA), the solution was increased to a volume of 40 µl with dH_2_O. Reverse transcription was performed at 37°C for 15 minutes, followed by an inactivation reaction at 85°C for 5 seconds. The resulting cDNA was stored at −20°C until use.

### Sample Analysis

Each cDNA was diluted using ddH2O to 1000 ul and mixed with 1 ml 2× SYBR Premix Ex Taq™ (TAKARA, Dalian, China). An average 20 µl of the mixture was added to each well of the 96 well PCR array except GDC control. The sealed PCR plate was loaded in Roche LightCycler 480 II instrument. The cycling condition is as following: 95°C for 5 min, 45 cycles at 95°C for 10 s, 60°C for 10 s and 72°C for 10 s. Then, dissociation curves (DC) and melting temperatures (Tm) were recorded. Melting curve analysis was performed at 95°C for 5 s, 65°C for 1 min and 95°C for 0 sec. Relative changes in gene expression were calculated using the △△Ct (threshold cycle) method. Here, △△Ct = (Ct target-sample - Ct ref-sample ) - (Ct target-control - Ct ref-control). Fold change values were calculated using the formula as follows: expression fold changes  =  Target gene expression level of sample/Target gene expression level of control 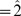
(−△△Ct).
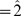


### Statistical Analysis

SPSS 13.0 was used for data analysis. All results are presented as mean±SD unless otherwise specified. Baseline data were compared by a one-way analysis of variance (ANOVA) between groups. The T-test was used for gene comparison between two groups. All p-values were two tailed, and a value <0.05 was considered to be statistically significant.

## Supporting Information

Figure S1 **Sensitivity evaluation of PCR array.** The PCR array system achieved greater than 80 percent positive calls with all samples, and 5 out of 9 over 90% positive calls, 8 out of 9 over 85%.
(DOCX)

Figure S2 **Specificity evaluation of PCR array.** Melting curve analysis shown that single peak could be obtained for each reaction which indicated the high specificity of PCR array.
(DOCX)

Figure S3 **Reproducibility evaluation of PCR array.** The bar repesents Ct value of PPC for each sample. The Cts of PPC in our experiments were 24±0.5 which demonstrated that high degree of plate-to-plate and run-to-run reproducibility could be obtained.
(DOCX)

Table S1 **Gene Table and Transcripts Detected by the Primers.**
(DOC)
