# Supplementary material for: A Pilot Trial Assessing Urinary Gene Expression Profiling with an mRNA Array for Diabetic Nephropathy
Source: PLoS One. 2012 May 18;7(5):e34824. doi: 10.1371/journal.pone.0034824 (PMC3356359; doi:10.1371/journal.pone.0034824)
Supplement: Figure S1 — Sensitivity evaluation of PCR array. The PCR array system achieved greater than 80 percent positive calls with all samples, and 5 out of 9 over 90% positive calls, 8 out of 9 over 85%. (DOCX) [file pone.0034824.s001.docx]

Supplemental file 1


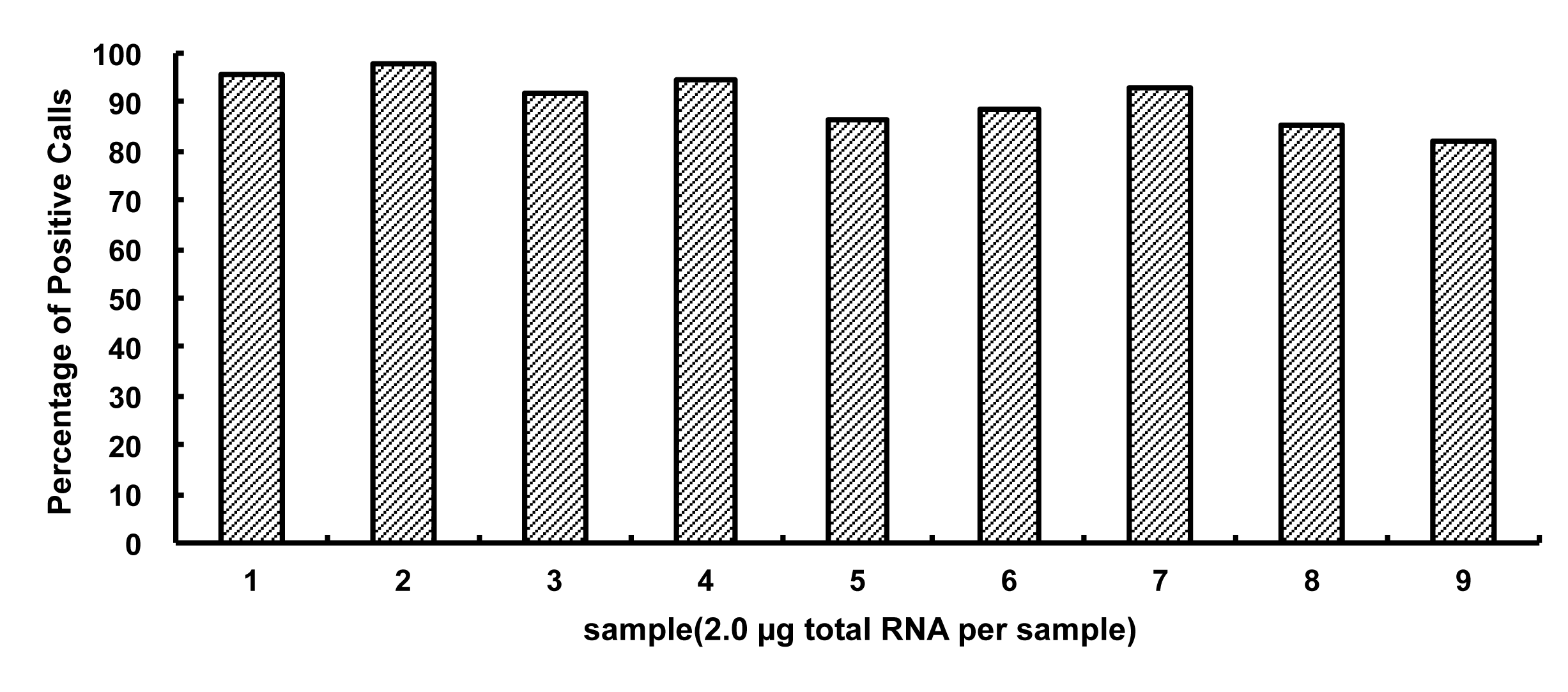


**Supplemental file 1. Sensitivity evaluation of PCR array.** The PCR array system achieved greater than 80 percent positive calls with all samples, and 5 out of 9 over 90% positive calls, 8 out of 9 over 85%.
