# Supplementary material for: A Pilot Trial Assessing Urinary Gene Expression Profiling with an mRNA Array for Diabetic Nephropathy
Source: PLoS One. 2012 May 18;7(5):e34824. doi: 10.1371/journal.pone.0034824 (PMC3356359; doi:10.1371/journal.pone.0034824)
Supplement: Figure S2 — Specificity evaluation of PCR array. Melting curve analysis shown that single peak could be obtained for each reaction which indicated the high specificity of PCR array. (DOCX) [file pone.0034824.s002.docx]

Supplemental file 2


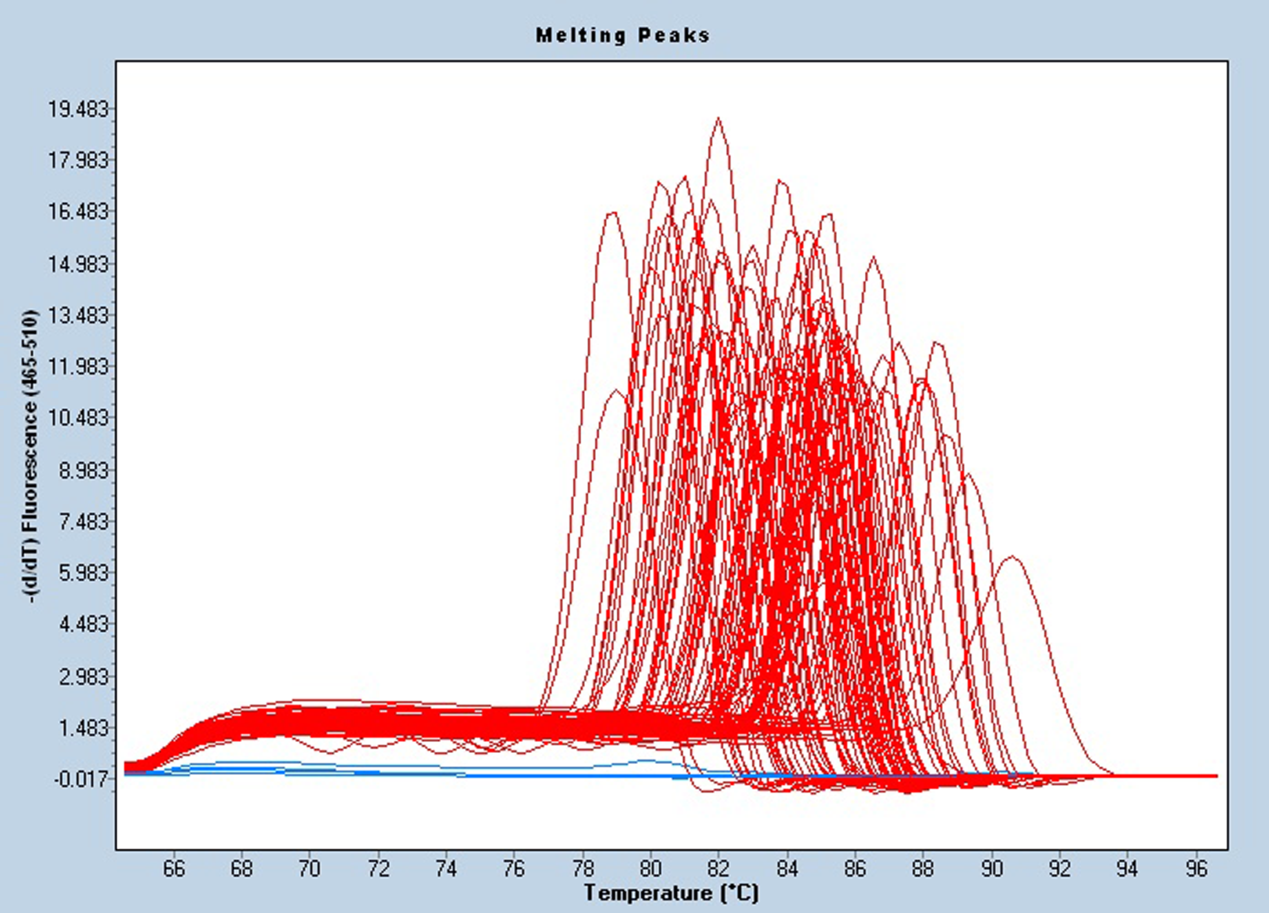


**Supplemental file 2. Specificity evaluation of PCR array.** Melting curve analysis shown that single peak could be obtained for each reaction which indicated the high specificity of PCR array.
