# Supplementary material for: A Pilot Trial Assessing Urinary Gene Expression Profiling with an mRNA Array for Diabetic Nephropathy
Source: PLoS One. 2012 May 18;7(5):e34824. doi: 10.1371/journal.pone.0034824 (PMC3356359; doi:10.1371/journal.pone.0034824)
Supplement: Figure S3 — Reproducibility evaluation of PCR array. The bar repesents Ct value of PPC for each sample. The Cts of PPC in our experiments were 24±0.5 which demonstrated that high degree of plate-to-plate and run-to-run reproducibility could be obtained. (DOCX) [file pone.0034824.s003.docx]

Supplemental file 3


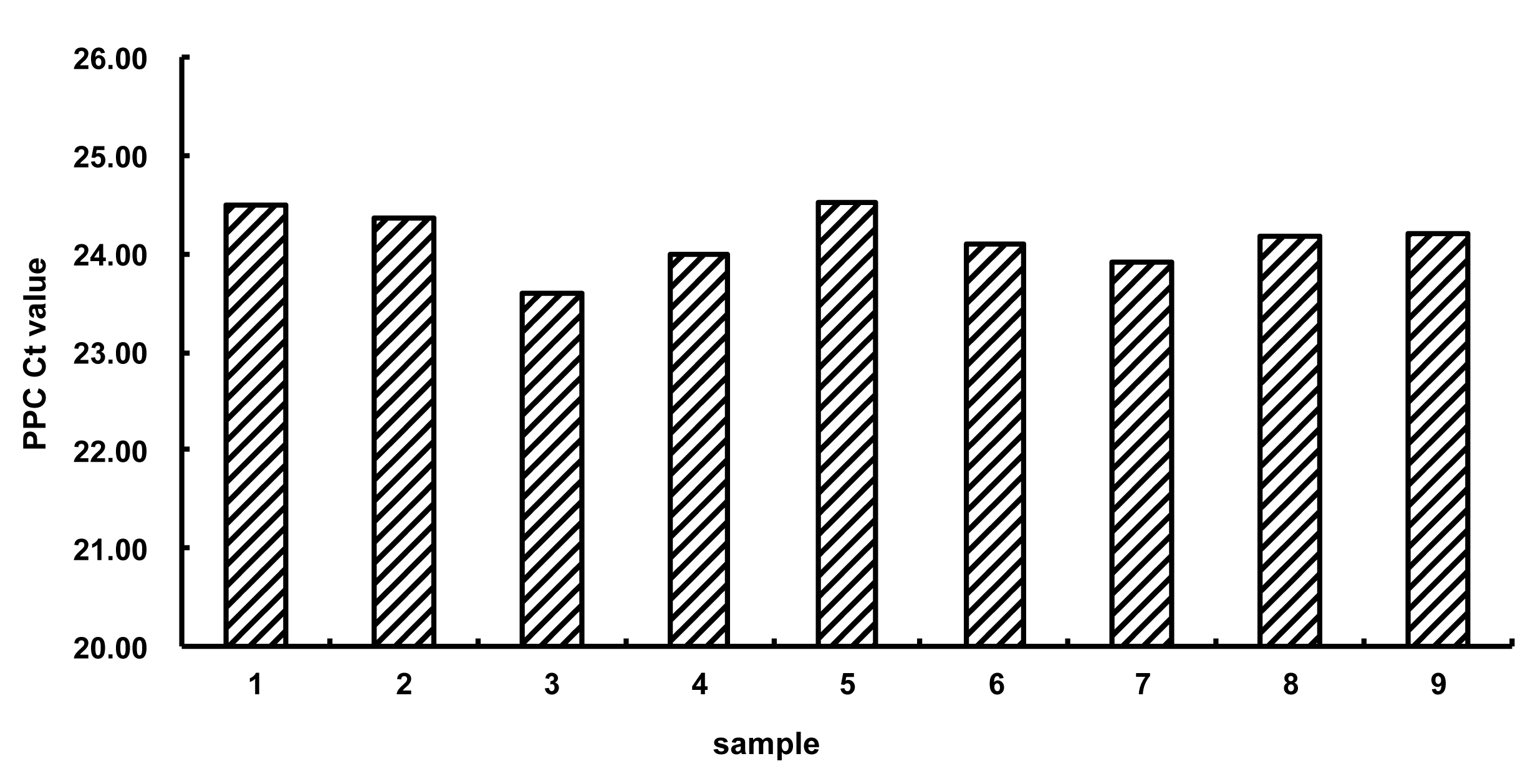


**Supplemental file 3. Reproducibility evaluation of PCR array.** The bar repesents Ct value of PPC for each sample. The Cts of PPC in our experiments were 24±0.5 which demonstrated that high degree of plate-to-plate and run-to-run reproducibility could be obtained.
