# Supplementary material for: A Pilot Trial Assessing Urinary Gene Expression Profiling with an mRNA Array for Diabetic Nephropathy
Source: PLoS One. 2012 May 18;7(5):e34824. doi: 10.1371/journal.pone.0034824 (PMC3356359; doi:10.1371/journal.pone.0034824)
Supplement: Table S1 — Gene Table and Transcripts Detected by the Primers. (DOC) [file pone.0034824.s004.doc]

Table S1. Gene Table and Transcripts Detected by the Primers

| **Symbol** | **GeneID as in PCR array** | **GeneID** | **Gene Name** | **Detected Transcripts of the gene** | **Detected all transcripts of the gene** |
| --- | --- | --- | --- | --- | --- |
| HGF | Gene1 | 3082 | hepatocyte growth factor | NM_000601.4 NM_001010931.1 NM_001010932.1 NM_001010933.1 NM_001010934.1 | yes |
| IGF1 | Gene2 | 3479 | insulin-like growth factor 1 | NM_000618.3 NM_001111283.1 NM_001111284.1 NM_001111285.1 | yes |
| CTGF | Gene3 | 1490 | connective tissue growth factor | NM_001901.2 | yes |
| VEGFC | Gene4 | 7424 | vascular endothelial growth factor C | NM_005429.2 | yes |
| VEGFB | Gene5 | 7423 | vascular endothelial growth factor B | NM_003377.3 | yes |
| PDGFB | Gene6 | 5155 | platelet-derived growth factor beta polypeptide | NM_002608.2 NM_033016.2 | yes |
| FGF23 | Gene7 | 8074 | fibroblast growth factor 23 | NM_020638.2 | yes |
| EGF | Gene8 | 1950 | epidermal growth factor | NM_001178130.1 NM_001178131.1 NM_001963.4 | yes |
| IL1A | Gene9 | 3552 | interleukin 1, α | NM_000575.3 | yes |
| IL1B | Gene10 | 3553 | interleukin 1, β | NM_000576.2 | yes |
| IL6 | Gene11 | 3569 | interleukin 6 | NM_000600.3 | yes |
| IL8 | Gene12 | 3576 | interleukin 8 | NM_000584.2 | yes |
| IL18 | Gene13 | 3606 | interleukin 18 | NM_001562.2 | yes |
| ICAM1 | Gene14 | 3383 | intercellular adhesion molecule 1 | NM_000201.2 | yes |
| VCAM1 | Gene15 | 7412 | vascular cell adhesion molecule 1 | NM_001078.2 NM_080682.1 | yes |
| CRP | Gene16 | 1401 | C-reactive protein, pentraxin-related | NM_000567.2 | yes |
| NPHS2 | Gene17 | 7827 | nephrosis 2, idiopathic, steroid-resistant (podocin) | NM_014625.2 | yes |
| CD2AP | Gene18 | 23607 | CD2-associated protein | NM_012120.2 | yes |
| ITGA3 | Gene19 | 3675 | integrin, α3 | NM_002204.2 NM_005501.2 | yes |
| ITGB1 | Gene20 | 3688 | integrin, β1 | NM_002211.3 NM_033668.2 NM_133376.2 | no |
| TJP1 | Gene21 | 7082 | tight junction protein 1 (zona occludens 1) | NM_003257.3 NM_175610.2 | yes |
| CDH3 | Gene22 | 1001 | cadherin 3, type 1, Gene P-cadherin | NM_001793.4 | yes |
| FAT1 | Gene23 | 2195 | FAT tumor suppressor homolog 1 | NM_005245.3 | yes |
| SYNPO | Gene24 | 11346 | synaptopodin | NM_001109974.2 NM_001166208.1 NM_001166209.1 NM_007286.5 | yes |
| ACTN4 | Gene25 | 81 | actinin, α4 | NM_004924.3 | yes |
| PODXL | Gene26 | 5420 | podocalyxin-like | NM_001018111.2 NM_005397.3 | yes |
| PODXL2 | Gene27 | 50512 | podocalyxin-like 2 | NM_015720.2 | yes |
| MMP2 | Gene28 | 4313 | matrix metallopeptidase 2 | NM_001127891.1 NM_004530.4 | yes |
| MMP9 | Gene29 | 4318 | matrix metallopeptidase 9 | NM_004994.2 | yes |
| TIMP2 | Gene30 | 7077 | TIMP metallopeptidase inhibitor 2 | NM_003255.4 | yes |
| TIMP1 | Gene31 | 7076 | TIMP metallopeptidase inhibitor 1 | NM_003254.2 | yes |
| COL3A1 | Gene32 | 1281 | collagen, type III, α1 | NM_000090.3 | yes |
| COL4A1 | Gene33 | 1282 | collagen, type IV, α1 | NM_001845.4 | yes |
| CDH1 | Gene34 | 999 | cadherin 1, type 1, E-cadherin (epithelial) | NM_004360.3 | yes |
| VIM | Gene35 | 7431 | vimentin | NM_003380.3 | yes |
| S100A4 | Gene36 | 6275 | S100 calcium binding protein A4 | NM_002961.2 NM_019554.2 | yes |
| DES | Gene37 | 1674 | desmin | NM_001927.3 | yes |
| CDH2 | Gene38 | 1000 | cadherin 2, type 1, N-cadherin | NM_001792.3 | yes |
| TWIST1 | Gene39 | 7291 | twist homolog 1 | NM_000474.3 | yes |
| SNAI1 | Gene40 | 6615 | snail homolog 1 | NM_005985.3 | yes |
| SNAI2 | Gene41 | 6591 | snail homolog 2 | NM_003068.3 | yes |
| LEF1 | Gene42 | 51176 | lymphoid enhancer-binding factor 1 | NM_001130713.2 NM_001130714.2 NM_001166119.1 NM_016269.4 | yes |
| ETS1 | Gene43 | 2113 | v-ets erythroblastosis virus E26 oncogene homolog | NM_001143820.1 NM_001162422.1 NM_005238.3 | yes |
| ILK | Gene44 | 3611 | integrin-linked kinase | NM_001014794.1 NM_001014795.1 NM_004517.2 | yes |
| SMAD1 | Gene45 | 4086 | SMAD family member 1 | NM_001003688.1 NM_005900.2 | yes |
| SMAD2 | Gene46 | 4087 | SMAD family member 2 | NM_001003652.2 NM_001135937.1 NM_005901.4 | yes |
| SMAD3 | Gene47 | 4088 | SMAD family member 3 | NM_001145102.1 NM_001145103.1 NM_001145104.1 NM_005902.3 | yes |
| SMAD4 | Gene48 | 4089 | SMAD family member 4 | NM_005359.5 | yes |
| SMAD7 | Gene49 | 4092 | SMAD family member 7 | NM_001190821.1 NM_001190822.1 NM_001190823.1 NM_005904.3 | yes |
| BMP7 | Gene50 | 655 | bone morphogenetic protein 7 | NM_001719.2 | yes |
| LAMC2 | Gene51 | 3918 | laminin, γ2 | NM_005562.2 NM_018891.2 | yes |
| LAMA5 | Gene52 | 3911 | laminin, α5 | NM_005560.3 | yes |
| CCL5 | Gene53 | 6352 | chemokine (C-C motif) ligand 5 | NM_002985.2 | yes |
| CP | Gene54 | 1356 | ceruloplasmin (ferroxidase) | NM_000096.3 | yes |
| AMBP | Gene55 | 259 | α-1-microglobulin/bikunin precursor | NM_001633.3 | yes |
| B2M | Gene56 | 567 | β-2-microglobulin | NM_004048.2 | yes |
| UMOD | Gene57 | 7369 | uromodulin | NM_001008389.1 NM_003361.2 | yes |
| PPARG | Gene58 | 5468 | peroxisome proliferator-activated receptorγ | NM_005037.5 NM_015869.4 NM_138711.3 NM_138712.3 | yes |
| RAGE | Gene59 | 5891 | renal tumor antigen | NM_014226.1 | yes |
| REN | Gene60 | 5972 | renin | NM_000537.3 | yes |
| ACE | Gene61 | 1636 | angiotensin I converting enzyme | NM_001178057.1 | no |
| ACE2 | Gene62 | 59272 | angiotensin I converting enzyme | NM_021804.2 | yes |
| HAVCR1 | Gene63 | 26762 | hepatitis A virus cellular receptor 1 | NM_001099414.1 NM_001173393.1 NM_012206.2 | yes |
| LCN2 | Gene64 | 3934 | lipocalin 2 | NM_005564.3 | yes |
| NOTCH1 | Gene65 | 4851 | Notch homolog 1, translocation-associated | NM_017617.3 | yes |
| NOTCH2 | Gene66 | 4853 | Notch homolog 2 | NM_024408.2 | yes |
| NOTCH3 | Gene67 | 4854 | Notch homolog 3 | NM_000435.2 | yes |
| NOTCH4 | Gene68 | 4855 | Notch homolog 4 | NM_004557.3 | yes |
| JAG1 | Gene69 | 182 | jagged 1 | NM_000214.2 | yes |
| JAG2 | Gene70 | 3714 | jagged 2 | NM_002226.3 NM_145159.1 | yes |
| MAPK8 | Gene71 | 5599 | mitogen-activated protein kinase 8 | NM_002750.2 NM_139046.1 NM_139047.1 NM_139049.1 | yes |
| TGFB1 | Gene72 | 7040 | transforming growth factor, β1 | NM_000660.4 | yes |
| NPHS1 | Gene73 | 4868 | nephrosis 1, congenital, Finnish type (nephrin) | NM_004646.3 | yes |
| TNF | Gene74 | 7124 | tumor necrosis factor (TNF superfamily, member 2) | NM_000594.2 | yes |
| CCL2 | Gene75 | 6347 | chemokine (C-C motif) ligand 2 | NM_002982.3 | yes |
| IFNG | Gene76 | 3458 | interferon, γ | NM_000619.2 | yes |
| WT1 | Gene77 | 7490 | Wilms tumor 1 | NM_000378.4 NM_001198551.1 NM_001198552.1 NM_024424.3 NM_024426.4 | yes |
| α-SMA | Gene78 | 59 | ACTA2 actin, α2, smooth muscle | NM_001141945.1 NM_001613.2 | yes |
| SERPINE1 | Gene79 | 5054 | serpin peptidase inhibitor, clade E | NM_000602.3 NM_001165413.1 | yes |
| FN1 | Gene80 | 2335 | fibronectin 1 | NM_002026.2 NM_054034.2 NM_212474.1 NM_212476.1 NM_212478.1 NM_212482.1 | no |
| FABP1 | Gene81 | 2168 | fatty acid binding protein 1, liver | NM_001443.1 | yes |
| KRT18 | Gene82 | 3875 | keratin 18 | NM_000224.2 NM_199187.1 | yes |
| RBP4 | Gene83 | 5950 | retinol binding protein 4, plasma | NM_006744.3 | yes |
| TF | Gene84 | 7018 | transferrin | NM_001063.3 | yes |
| MAPK14 | Gene85 | 1432 | mitogen-activated protein kinase 14 | NM_001315.2 NM_139012.2 NM_139013.2 NM_139014.2 | yes |
| NFKB1 | Gene86 | 4790 | nuclear factor of κ light polypeptide gene enh | NM_001165412.1 NM_003998.3 | yes |
| AGT | Gene87 | 183 | angiotensinogen (serpin peptidase inhibitor, clade) | NM_000029.3 | yes |
| COL5A2 | Gene88 | 1290 | collagen, type V, α2 | NM_000393.3 | yes |
